# Preserving mitochondria to treat hypertrophic cardiomyopathy: From rare mitochondrial DNA mutation to heart failure therapy?

**DOI:** 10.1172/JCI171965

**Published:** 2023-07-17

**Authors:** Abhinav Diwan

**Affiliations:** 1 Department of Medicine,; 2 Department of Cell Biology and Physiology,; 3 Department of Obstetrics and Gynecology,; 4 Department of Neurology,; 5 Center for Cardiovascular Research, and; 6 Hope Center for Neurologic Disorders, Washington University School of Medicine, St. Louis, Missouri, USA.; 7 John Cochran VA Medical Center, St. Louis, Missouri, USA.

## Abstract

Hypertrophic cardiomyopathy and pathological cardiac hypertrophy are characterized by mitochondrial structural and functional abnormalities. In this issue of the *JCI*, Zhuang et al. discovered 1-deoxynojirimycin (DNJ) through a screen of mitochondrially targeted compounds. The authors described the effects of DNJ in restoring mitochondria and preventing cardiac myocyte hypertrophy in cellular models carrying a mutant mitochondrial gene, *MT-RNR2*, which is causally implicated in familial hypertrophic cardiomyopathy. DNJ worked via stabilization of the mitochondrial inner-membrane GTPase OPA1 and other, hitherto unknown, mechanisms to preserve mitochondrial crista and respiratory chain components. The discovery is likely to spur development of a class of therapeutics that restore mitochondrial health to prevent cardiomyopathy and heart failure.

## Preserving mitochondria to counter cardiomyopathy and heart failure

Understanding the mechanisms by which genetic changes cause human disease holds tremendous promise for developing therapies. This process is exemplified by the discovery of cholesterol regulation via the LDL receptor (1) that led to development of statins and PCSK9 inhibitors to treat hypercholesterolemia for lowering cardiovascular risk. As another example, the development of myosin modulators to prevent cardiac myocyte hypercontractility with sarcomeric mutations (2) now provides a treatment for hypertrophic cardiomyopathy. In this issue of the *JCI*, Zhuang and authors discovered a small molecule by screening a library of mitochondrially-targeted compounds that preserved mitochondrial membrane potential in cells carrying defective mitochondria due to the *MT-RNR2* (3) mutation, which was previously described in a multigenerational family with hypertrophic cardiomyopathy (4). These studies raise hopes for developing a class of therapies that preserve healthy mitochondria to counter cardiomyopathy and heart failure, where mitochondrial dysfunction is triggered by diverse pathologies.

Hypertrophic cardiomyopathy, a condition that affects approximately one in 500 individuals with substantially increased premature mortality and morbidity, is generally recognized as a genetic disease with sarcomeric gene mutations transmitted in an autosomal dominant fashion as the most common etiology (5). Intriguingly, an emerging body of literature has probed maternal inheritance of hypertrophic cardiomyopathy and implicated mitochondrial DNA mutations in the pathogenesis of hypertrophic cardiomyopathy. These include mutations in mitochondrial tRNA (specifically Leu (UUR) in *MT-TL1*), in ND5 (12338T>C, a mitochondrial gene encoding for NADH dehydrogenase 5, a part of complex I), and the specific mutation in mitochondrial 16sRNA (2336T>C in *MT-RNR2* (6)), which is the focus of this chemical screen (3). The latter mutation impaired mitochondrial membrane potential in cybrids (hybrid cells engineered to carry patient-derived mitochondria) and induced mitochondrial defects in induced pluripotent stem cell (iPS)-derived cardiac myocytes (3) that were reversed by 1-deoxynojirimycin (DNJ). Zhuang, et al. presented a comprehensive dataset in cell-culture studies to demonstrate the efficacy of DNJ in preventing cellular hypertrophy and restoring mitochondrial structure, membrane potential, oxidative phosphorylation, and ATP production (3).

## Disease modifying agents for hypertrophic cardiomyopathy

The next steps will require preclinical testing of DNJ (3) in relevant animal models to determine its efficacy in modifying myocardial pathology, such as hypertrophy, fibrosis, and disarray, in hypertrophic cardiomyopathy. Indeed, there is an urgent need for developing disease-modifying approaches, as the current mainstays of therapies for hypertrophic cardiomyopathy (which include negative inotropic agents such as beta blockers, nondihydropyridine calcium channel antagonists, and disopyramide) reduce dynamic left ventricular outflow tract obstruction without altering disease progression (i.e., the lack of effect on left ventricular hypertrophy) (5). Moreover, while preclinical studies demonstrated a robust effect of the myosin modulator, mavacamten, in preventing and ameliorating left ventricular hypertrophy in mouse models (2), clinical trials in patients with established disease have primarily demonstrated symptomatic relief in patients with dynamic left ventricular outflow tract obstruction and not in those with non-obstructive forms of hypertrophic cardiomyopathy (7). As an exciting development, recent studies demonstrated the feasibility of in vivo genome editing with adenine-base editor and CRISPR technology to correct myosin heavy chain gene mutations, which successfully prevented development of hypertrophy in iPS-derived cardiac myocytes and attenuated ventricular remodeling and fibrosis in mouse models (8, 9). The development of mitochondrial genome editing tools with engineered deaminases to effect C-to-T or A-to-G changes offers an analogous tool for preventing disease in patients carrying pathogenic mitochondrial DNA mutations, but major challenges remain (10). Indeed, while the vast majority of mitochondrial proteins are encoded by nuclear genes and are amenable to genetic correction, mitochondrial DNA poses challenges to editing. Correction of mitochondrial DNA mutations is difficult because the mitochondrial genome is comprised of small, circular, double-stranded DNA (approximately 16.5 Kb in size, which encodes for 13 proteins, 22 tRNAs, and two rRNAs (11)), and is present in multiple copies per cell that may be similar (homoplasmy) or carry different variants (heteroplasmy). Alternatively, mitochondrial replacement offers another therapeutic paradigm for treating mitochondrial diseases and has been actively pursued since its first successful demonstration for assisted human reproductive technology in human oocytes (12).

## Mechanisms of action for DNJ

To probe the mechanisms of action for DNJ, Zhuang et al. performed affinity purification and identified more than 900 interacting proteins (3). Through focusing on mitochondrial proteins, the authors implicated OPA1, which is encoded by a nuclear gene (*OPA1*), as the target. They also demonstrated that DNJ stabilized OPA1 oligomers to increase ATP generation, which is consistent with its role as a master regulator of crista formation and functional assembly of respiratory chain complexes (13). It remains unclear if the OPA1 interaction is the primary mechanism underlying the benefits of DNJ. While knockdown of OPA1 prevented the beneficial effects of DNJ, it also worsened the mitochondrial phenotypes in *MT-RNR2* mutant cells in the control-treated group (3), analogous to the effects of OPA1 knockdown in WT cells (13). Therefore, it is possible that OPA1 knockdown induced severe mitochondrial impairment in the setting of *MT-RNR2* mutation beyond a point of no return. Moreover, other mitochondrial proteins, including ATP synthase protein 8 (encoded by *MT-ATP8*), cytochrome c oxidase subunit 2 (encoded by *MT-COX2*), and ATP synthase subunit a (encoded by *MT-ATP6*), were also isolated as DNJ interactors (3). These binding partners may play a role as chaperones in stabilizing multiple mitochondrial proteins or in promoting mitochondrial translation by stabilizing ribosomal 16s RNA — encoded by the mutant MT-RNR2 (6) — a premise that is supported by the preservation of respiratory chain complexes with DNJ treatment (3).

The efficacy of mitochondrial preservation in preventing hypertrophic cardiomyopathy-related phenotypes (3) begs the question of whether hypertrophic cardiomyopathy from diverse etiologies is a disease of mitochondrial energetic insufficiency that may benefit from mitochondrial preservation strategies. Intriguingly, mitochondrial structural and functional abnormalities were noted in septal myomectomy tissue from patients with hypertrophic cardiomyopathy secondary to sarcomeric gene mutations with preserved left ventricular systolic function (14). The mitochondria were noted to be smaller in size with cristal rarefaction, and there was evidence of reduced oxidative phosphorylation with increased levels of reactive oxygen species. This finding was accompanied by a reduction in markers of mitophagy, a selective autophagy pathway for lysosomal degradation of damaged and dysfunctional mitochondria, and organ-level abnormalities in carbohydrate and lipid metabolism with ATP deficiency (14). Moreover, abnormalities in nonselective autophagy have also been documented in myocardial tissue from patients with mutations in *MYBPC3*, encoding cardiac myosin-binding protein C, which represent the most common genetic etiology linked with hypertrophic cardiomyopathy (15). Interestingly, rescuing impaired myocardial autophagy, reduced cardiac hypertrophy, and attenuated left ventricular systolic dysfunction in a knock-in mouse model bearing another hypertrophic cardiomyopathy-linked *MYBPC3* mutation (15). A role for the autophagy pathway and the endosomal sorting complexes required for transport (ESCRT) complex, which sorts polyubiquitinated proteins for lysosomal degradation, was also highlighted by the discovery of massive cardiac hypertrophy and sudden death in mice with striated muscle-specific ablation of vacuolar protein sorting 34 (Vps34) (16). VPS34 is a class III phosphoinositide 3-kinase that generates the membrane lipid phosphatidylinositol 3-phosphate (PI3P) to facilitate endosomal and autophagosome membrane formation; and VPS34 levels are reduced in myocardium from patients with hypertrophic cardiomyopathy linked to sarcomeric mutations (16). Whether mitochondrial preservation strategies can bypass impairment in non-selective autophagy or specific impairments in mitophagy to ameliorate hypertrophic cardiomyopathy remains to be explored.

Conceivably, DNJ may stimulate mitophagy to remove abnormal mitochondria as a mechanism that contributes to restoration of mitochondrial structure and function, a possibility that requires experimental examination. Mitophagy is robustly active in the unstressed myocardium (17) and stimulation of mitophagy restores normal mitochondria to ameliorate left ventricular hypertrophy, fibrosis, and dysfunction in a mouse model of pressure overload-induced hypertrophy (18). Canonical mitophagy requires normally functioning lysosomes and Mit/Tfe family of master transcriptional regulators, which control autophagy and lysosome biogenesis (19). Interestingly, multiple lysosomal storage diseases (LSDs) characterized by lysosome dysfunction, phenocopy hypertrophic cardiomyopathy, including the development of heart failure, arrhythmias, and premature death (20). These LSDs include Fabry’s disease, provoked by mutations in *GLA* gene resulting in insufficiency of α-galactosidase A; Pompe’s disease, which results from mutations in *GAA* gene that encodes for α-glucosidase; and Danon disease, which results from mutations in *LAMP2* that encodes for a lysosomal membrane protein. Intriguingly, Zhuang et al. demonstrate that DNJ bound to α-glucosidase (3). While α-glucosidase did not appear to be required for its beneficial effects on mitochondria, the effects of DNJ on lysosome function will require further examination in animal models. Indeed, treatment with 1-deoxygalactonojirimycin (approved as Migalastat), which is a stereoisomer of DNJ, resulted in reduced left ventricular hypertrophy in patients with Fabry’s disease (21). 1-deoxygalactonojirimycin facilitates transport of α-galactosidase A to lysosomes via a proposed chaperone-like activity. Another related compound, N-butyldeoxynojirimycin (Miglustat), was also shown to bind and stabilize recombinant α-glucosidase (22), likely due to structural similarity with glucose. Interestingly, a combination of N-butyldeoxynojirimycin and α-glucosidase enzyme replacement reduced glycogen accumulation with correction of autophagic defects in a mouse model of Pompe’s disease (22) and improved functional outcomes in a clinical trial in patients with late-onset Pompe’s disease (23).

**Figure 1:**
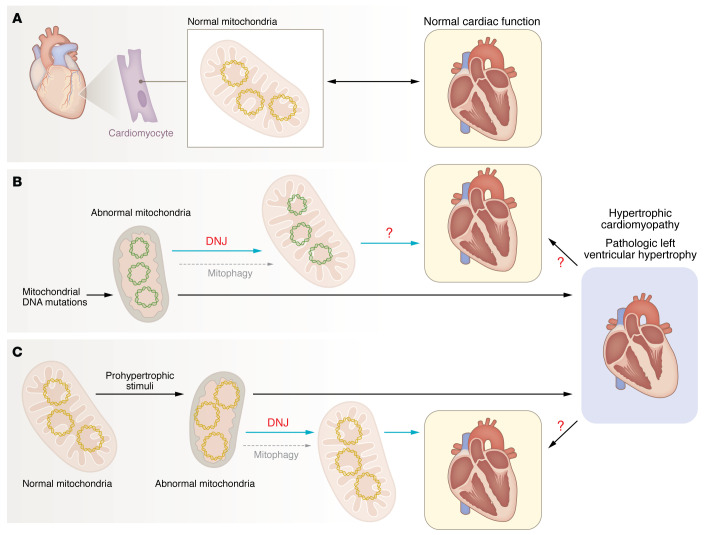
Restoration of mitochondrial structure and function ameliorates pathologic cardiac hypertrophy and preserves normal function. (**A**) Mitochondria in cardiac cells support high energy demands for the function of a healthy heart. (**B**) Mutations in mitochondrial DNA can provoke mitochondrial fragmentation with loss of cristae and trigger hypertrophic cardiomyopathy. Treatment with DNJ restores mitochondrial structure with normalization of cristae, despite presence of the mitochondrial DNA mutation, to prevent cardiac myocyte hypertrophy. It remains unknown whether treatment with DNJ will prevent development of hypertrophic cardiomyopathy or reverse established disease. Whether DNJ stimulates mitophagy to restore normal mitochondria also remains to be determined. (**C**) Prohypertrophic stimuli, such as hemodynamic overload, can also result in loss of mitochondrial function, independent of mutations in mitochondrial DNA, and result in pathologic cardiac hypertrophy and cardiomyopathic decompensation. Treatment of mice with angiotensin II as a model to mimic the effects of hemodynamic overload induces mitochondrial structural and functional defects, including mitochondrial fragmentation with loss of cristae with development of pathologic cardiac hypertrophy and left ventricular systolic dysfunction. Notably, treatment with DNJ restores mitochondrial structure and function to attenuate hypertrophy and prevents angiotensin II-induced left ventricular systolic dysfunction. Whether DNJ will reverse established pathologic hypertrophy and cardiomyopathy remains unknown.

Zhuang and authors extended their findings to a mouse model of angiotensin-II–induced pathologic hypertrophy and demonstrated that DNJ prevented pathologic hypertrophy and fibrosis and preserved mitochondrial structure, electron transport chain subunits, and ATP production (3). DNJ also restored OPA1 oligomers and mitochondrial respiratory chain complex proteins in this setting (3). Interestingly, OPA1 overexpression is sufficient to induce physiologic left ventricular hypertrophy with increased exercise capacity in mice (24); and transgenic overexpression of PERM1, a protein that interacts with MICOS complex to stabilize cristae (13), preserves mitochondrial oxidative phosphorylation and prevents development of pathologic cardiac hypertrophy and decompensation with pressure overload (25). These results raise the exciting possibility that restoring mitochondrial health may prevent and/or treat pathologic cardiac hypertrophy that is provoked by increased hemodynamic load and other etiologies characterized by mitochondrial defects (Figure 1). In this context, it also relevant to note that while Zhuang et al. demonstrated cell-autonomous effects of DNJ in cardiac myocytes (3), the contribution of systemic effects of DNJ administration, including modulation of inter-cellular and inter-organ cross-talk, remains to be explored.

Notwithstanding the need for clarification of mechanisms, the identification of DNJ provides a shot in the arm for the development of drugs to restore normal mitochondria in the treatment of pathologic cardiac hypertrophy and heart failure.

## Version 1 07/17/2023

Electronic publication
